# Modeled PFOA Exposure and Coronary Artery Disease, Hypertension, and High Cholesterol in Community and Worker Cohorts

**DOI:** 10.1289/ehp.1307943

**Published:** 2014-09-26

**Authors:** Andrea Winquist, Kyle Steenland

**Affiliations:** Environmental Health Department, Rollins School of Public Health, Emory University, Atlanta, Georgia, USA

## Abstract

Background: Several previous studies, mostly cross-sectional, have found associations between perfluorooctanoic acid (PFOA) and high cholesterol levels, but studies of hypertension and heart disease have had inconsistent findings.

Objectives: In this study we examined the association between modeled PFOA exposure and incident hypertension, hypercholesterolemia, and coronary artery disease among workers at a Mid-Ohio Valley chemical plant that used PFOA, and residents of the surrounding community.

Methods: Community- and worker-cohort participants completed surveys during 2008–2011 covering demographics, health-related behaviors, and medical history. Cox proportional hazard models, stratified by birth year, modeled the hazard of each outcome (starting at 20 years of age) as a function of retrospective serum PFOA concentration estimates (generated through fate, transport and exposure modeling), controlling for sex, race, education, smoking, alcohol use, body mass index, and diabetes.

Results: Among 32,254 participants (28,541 community; 3,713 worker), 12,325 reported hypertension with medication, 9,909 reported hypercholesterolemia with medication, and 3,147 reported coronary artery disease (2,550 validated). Hypercholesterolemia incidence increased with increasing cumulative PFOA exposure (sum of yearly serum concentration estimates), most notably among males 40–60 years of age. Compared with the lowest exposure quintile (< 142 ng/mL-years), hazard ratios for subsequent quintiles (ng/mL-years: 142 to < 234; 234 to < 630; 630 to < 3,579; ≥ 3,579) were 1.24, 1.17, 1.19, and 1.19 overall and 1.38, 1.32, 1.31, and 1.44 among men 40–60 years of age. There was no apparent association between PFOA exposure and hypertension or coronary artery disease incidence.

Conclusions: Higher PFOA exposure was associated with incident hypercholesterolemia with medication, but not with hypertension or coronary artery disease.

Citation: Winquist A, Steenland K. 2014. Modeled PFOA exposure and coronary artery disease, hypertension, and high cholesterol in community and worker cohorts. Environ Health Perspect 122:1299–1305; http://dx.doi.org/10.1289/ehp.1307943

## Background

Perfluorooctanoic acid (PFOA) is a human-made 8-carbon perfluorinated compound used as an emulsifier in the manufacture of polytetrafluoroethylene (e.g., Teflon®) and other fluoropolymers (Lau et al. 2007). It is not easily broken down and persists in the environment (Lau et al. 2007). In humans, PFOA has a half-life of approximately 2.3–3.4 years (Bartell et al. 2010; Olsen et al. 2007). In the U.S. National Health and Nutrition Examination Survey (NHANES), PFOA was found in the blood of > 99% of U.S. adults during 1999–2008, despite reductions in PFOA emissions since 1999 (Kato et al. 2011). Human exposure in the general population is thought to occur through drinking water, house dust, food (including migration from packaging), and air (Fromme et al. 2009; Lau et al. 2007). Exposures at levels higher than the general population occur in workers and populations living near facilities using PFOA (Lau et al. 2007; Steenland et al. 2010a).

The relationship between PFOA and coronary artery disease is of concern because PFOA has been reported to be associated with several coronary artery disease risk factors, including hypertension (Min et al. 2012), higher serum cholesterol concentrations (Costa et al. 2009; Eriksen et al. 2013; Fisher et al. 2013; Fitz-Simon et al. 2013; Frisbee et al. 2010; Nelson et al. 2010; Olsen et al. 2003; Sakr et al. 2007a, 2007b; Steenland et al. 2009), higher serum uric acid levels (Geiger et al. 2013; Shankar et al. 2011; Steenland et al. 2010b), and higher serum homocysteine levels (Min et al. 2012). However, studies directly examining the relationship between PFOA and coronary artery disease have had mixed findings. Mortality studies conducted among occupational cohorts (including part of the cohort in this study) have not found associations between PFOA exposure and ischemic heart disease mortality (Leonard et al. 2008; Lundin et al. 2009; Sakr et al. 2009; Steenland and Woskie 2012). One cross-sectional study of PFOA exposure in the general U.S. population, using NHANES data (1999–2000, 2003–2004, and 2005–2006), also did not find an association with ischemic heart disease (defined as coronary heart disease, angina, and/or heart attack) among participants ≥ 20 years of age (Melzer et al. 2010), whereas a second study using NHANES data (1999–2000 and 2003–2004) did find an association with cardiovascular disease (defined as coronary artery disease, heart attack, or stroke) among participants ≥ 40 years of age (Shankar et al. 2012).

The present study is the first to longitudinally examine associations between PFOA exposure and hypercholesterolemia, hypertension, and coronary artery disease incidence among a population with high PFOA exposure. The population included workers at a chemical plant that used PFOA and residents of the surrounding community who were exposed to PFOA through contaminated drinking water. PFOA had been released from the chemical plant into the air, soil, and water starting in 1951 (Paustenbach et al. 2007). A class action law suit, filed in 2001–2002, alleged health damage due to PFOA exposure in the surrounding community. A settlement of the lawsuit led to a survey (the “C8 Health Project”), conducted in 2005–2006, of people who had been exposed for at least 12 months (through their primary residence, workplace, or school) to water in six water districts with PFOA contamination (Frisbee et al. 2009). The C8 Health Project survey included questions about medical history (with medical records validation of some conditions) and measurement of PFOA serum concentrations. The lawsuit settlement also formed the C8 Science Panel, a group of epidemiologists charged with determining whether PFOA (also called C8) was linked with any human diseases (http://www.c8sciencepanel.org). This study was conducted by the C8 Science Panel.

## Methods

*Cohort recruitment and survey administration*. This study included a community cohort and a worker cohort, which were combined for some analyses and also considered separately. The methods for cohort recruitment and survey administration have been previously described (Winquist et al. 2013). Community cohort participants were recruited among 40,145 C8 Health Project participants who were ≥ 20 years of age and consented to be contacted by the C8 Science Panel for further studies. Worker cohort participants were recruited from an occupational cohort, formed for previous mortality studies (Leonard et al. 2008; Sakr et al. 2009), that included 6,026 people who worked at the chemical plant during 1948–2002 (of whom 2,090 were also in the community cohort). Study participants were asked to complete questionnaires during two survey rounds (2008–2010 and 2010–2011; see Supplemental Material, Figure S1), covering demographics, health-related behaviors, and lifetime personal history of various medical diagnoses. Some participants completed a questionnaire during both survey rounds, and some during only one round. Proxy respondents were sought for people who were deceased or too ill to respond themselves. Participants gave informed consent before study participation. This study was approved by the Emory University Institutional Review Board.

At least one study questionnaire was completed during 2008–2011 for 32,712 (81.5%) of the 40,145 people in the community cohort target population (of whom 4,152 were excluded from the community cohort because they worked at the plant), and for 4,391 (72.9%) of the 6,026 people in the worker cohort target population. People in the community cohort also in the worker cohort were included only in the worker cohort. Retrospective modeled serum estimates were available for 28,541 (99.9%) community cohort participants and 3,713 (84.6%) worker cohort participants.

*Case definitions*. On the 2008–2011 surveys, participants were asked whether they had ever been told by a doctor or other health professional that they had high blood pressure, high cholesterol, or heart disease. Women were told to report hypertension only outside of pregnancy. Type of heart disease was also asked; choices included angina, arrhythmia, valve disease, heart attack, coronary artery disease, or “other” (asked to specify in a free text field). Responses in “other” fields were reviewed and classified. Reports (through selection of a listed option or through the text field) of angina, coronary atherosclerosis, coronary artery disease (including blocked arteries, bypass surgery, or stents), heart attack, or cardiac ischemia were considered to be reports of coronary artery disease for this analysis. For each reported condition, participants were asked the age at diagnosis. Participants reporting hypertension and high cholesterol were asked whether they were currently taking prescription medication for these conditions. Participants who reported hypertension or high cholesterol without medication during the first survey round were asked again about current medication use during the second survey round. If a proxy respondent reported hypertension or high cholesterol, the proxy was asked whether the person took prescription medication for the condition. Participants reporting heart disease were asked to consent for review of their medical records. Medical records were requested from the identified providers and were reviewed by trained medical record abstractors.

For this analysis, we analyzed incident hypercholesterolemia or hypertension cases reporting current prescription medication on the 2008–2011 surveys. We restricted cases of hypercholesterolemia and hypertension to those with prescription medication use as a way of identifying clinically important conditions. The onset age used in the analysis for these conditions was the date of first diagnosis, not the date of first medication use. People who reported hypertension or hypercholesterolemia without prescription medication use were excluded from the analysis. People diagnosed with persistent hypercholesterolemia or hypertension typically will take medication over their lifetime. However, we recognize that our case definition could have excluded some people who stopped medication before the survey, or who had been diagnosed but had not yet started medication use by the time of the survey. We think it is likely that these exclusions would be few, and there is no *a priori* reason why they would be associated with PFOA exposure.

Analyses for heart disease were restricted to cases for whom the medical record documented angina, coronary atherosclerosis, coronary artery disease, heart attack, or cardiac ischemia. We also accepted as valid any self-reported coronary artery disease cases (reported on the 2008–2011 surveys) that had been previously validated in the C8 Health Project survey (2005/2006).

*Exposure estimation*. Details of the exposure modeling are described elsewhere (Winquist et al. 2013). Briefly, an environmental fate and transport model was used to generate yearly estimates of PFOA concentrations in local air, surface water, and groundwater (Shin et al. 2011a). These concentrations were used, in combination with survey information relating to residential history, drinking-water sources, and water consumption rates, in a residential exposure model to estimate yearly PFOA intake rates (Shin et al. 2011b). Finally, the yearly intake estimates were used in a pharmacokinetic model to generate yearly PFOA serum concentration estimates (Shin et al. 2011b). For people in the worker cohort, job- and department-specific yearly PFOA serum concentration estimates were generated in an occupational model based on historical serum PFOA measurements, participants’ work histories, and knowledge of plant processes (Woskie et al. 2012). For people in the worker cohort, occupational exposure model estimates were used for the years when they worked at the plant if they were higher than the residential model estimates; if they were lower, the residential estimates were used. For years after a person stopped working at the plant, serum estimates were decayed 18% per year (based on a half-life of 3.5 years) (Olsen et al. 2007), until they reached a level predicted by the residential model. The Spearman’s rank correlation between modeled serum concentration estimates and serum concentrations measured in 2005–2006 (among 30,303 people with serum concentration measurements) was 0.71 (Winquist et al. 2013). For prospective analyses (starting 1 year after the age at the time of the C8 Health Project), estimates were calibrated to the measured serum PFOA concentrations using Bayesian calibration, with measurements weighted more heavily for estimates closer in time to the measurements. Retrospective analyses used uncalibrated estimates because of uncertainty about whether calibration would improve the accuracy of estimates for years far removed from the measurements.

*Data analysis*. We examined associations between PFOA serum concentration and the outcomes of interest using Cox proportional hazard models with age as the time scale and time-varying PFOA exposure measures. Retrospective analyses started at the later of age 20 years (to consider only adult disease) or the age in 1952 (the year after PFOA production started at the plant). Prospective analyses started at the participant’s age 1 year after enrollment in the C8 Health Project (2005–2006) or the age 1 year after 1 August 2006. Analyses ended at the earliest of the age at diagnosis of the condition of interest, death, or the last study questionnaire. Both types of analyses excluded those with a diagnosis of the condition of interest before the analysis start age and those missing a diagnosis age. We excluded people born before 1920 (*n* = 173) because of uncertain reliability of disease reporting in this group. Models for hypercholesterolemia and hypertension excluded people who reported the condition without current medication (*n* = 5,916 for hypercholesterolemia, and 2,470 for hypertension). Models for coronary artery disease excluded people who reported heart disease that was not validated coronary artery disease (597 reported coronary artery disease that was not validated, and 3,728 reported heart disease other than coronary artery disease). Models for coronary artery disease also excluded people without validated coronary artery disease but with coronary artery disease identified in mortality data (*n* = 75). All analyses were done using SAS version 9.2 (SAS Institute Inc., Cary, NC).

Because hypercholesterolemia, hypertension and coronary artery disease are chronic conditions that likely develop over time, our primary exposure metric was a measure of cumulative PFOA exposure, calculated as the sum of all yearly serum concentration estimates for a person through a given age. Because current PFOA serum concentrations could affect these outcomes more acutely, we also used the yearly serum PFOA concentration estimates (at time of case diagnosis or the corresponding age for noncases) in secondary analyses. Primary analyses considered the exposure by quintiles of the exposure metric, defined among the exposure estimates for cases at their diagnosis age. As a test for trend, we considered the log of the cumulative or yearly serum concentration estimates as a continuous variable.

There was some evidence of violation of the proportional hazards assumption for the cumulative and yearly exposure metrics in retrospective hypercholesterolemia analyses and in prospective hypertension analyses. Therefore, all analyses were performed both across all ages and separately by age strata (20–39, 40–59, and 60–79 years). All models were stratified by single-year birth year to tightly control for birth year. Models either included a term for the interaction between sex and age or considered sexes separately. Models also controlled for years of schooling [not time-varying; < 12 years, high school diploma/GED (General Educational Development), some college, or bachelor’s degree or higher], race (white vs. nonwhite or missing; Hispanic ethnicity was not considered because there were only 42 self-reported Hispanics in the study population), smoking (time-varying; current, former, none), smoking duration (time varying), smoking pack-years (time-varying linear term created by multiplying the self-reported number of packs smoked per day by the smoking duration to that point), regular alcohol consumption (time-varying; current, former, none), body mass index (BMI; at time of first study survey; underweight, normal, overweight, obese), and self-reported type 2 diabetes (time-varying according to reported age at diagnosis). Sensitivity analyses that did not control for self-reported type 2 diabetes gave very similar results (data not shown). Family history of coronary artery disease did not change estimates of exposure effect (data not shown) and was not included in models. Models for coronary artery disease did not control for hypercholesterolemia or hypertension because these outcomes were considered to potentially be in the causal pathway.

The primary analyses considered the combined cohorts (88% community, 12% workers). To assess the impact of combining the two cohorts (which likely had different exposure levels, different overall health status, and possibly different exposures to other environmental factors), we considered sensitivity analyses confined to the community cohort. To examine the extent to which results may have been affected by low exposures occurring before a person lived in the study area, and possible differential migration into the area by disease status, additional sensitivity analyses started at the first age at which each person was known to have qualified for one of the cohorts (by living in the study area for at least 1 year or working at the plant, referred to as the “qualifying year”). Additional sensitivity analyses considered myocardial infarction as an outcome (a subset of coronary artery disease cases), or were restricted to nonsmokers (to consider a group without another strong cardiovascular disease risk factor). To assess possible effect modification by calendar time, we considered models that ended the cohort follow-up (and hence the analysis) in varying calendar years, in 3-year intervals back to 1987 (before peak exposure).

## Results

*Cohort characteristics*. Characteristics of study participants overall and by cohort are shown in Table 1. In the combined cohort, 54% were female, 97% were of white race, 71% were overweight or obese, 52% reported ever smoking, and 14% reported ever having a type 2 diabetes diagnosis. The median follow-up duration was 32.6 years starting at the age in 1952 or age 20 years, and 4.4 years starting at the time of the C8 Health Project. The median PFOA serum concentration measured in 2005–2006 was 26.1 ng/mL. Median estimated serum PFOA concentrations (see Supplemental Material, Figure S2) were highest in the late 1990s and early 2000s (Winquist et al. 2013). Table 2 reports the number of people reporting the outcomes of interest, the number with reported current medication or medical records validation, and the number included in the analyses. The mean age at first diagnosis was 47 years for hypertension among those who reported prescription medication use, 50 years for hypercholesterolemia among those who reported prescription medication use, and 56 years for coronary artery disease among those for whom the condition was validated.

**Table 1:** Population characteristics [*n* (%) or ng/mL].

Characteristic	Community cohort only (*n*= 28,541)	Worker cohort only (*n *= 3,713)	Combined cohort (*n *= 32,254)
Female	16,602 (58)	758 (20)	17,360 (54)
Birth year
< 1920	114 (0.4)	59 (2)	173 (0.5)
1920–1939	3,742 (13)	727 (20)	4,469 (14)
1940–1959	11,547 (40)	1,814 (49)	13,361 (41)
≥ 1960	13,138 (46)	1,113 (30)	14,251 (44)
Education (4 missing)
< High school	3,026 (11)	37 (1)	3,063 (9)
High school diploma or GED	11,706 (41)	1,265 (34)	12,971 (40)
Some college	9,441 (33)	1,081 (29)	10,522 (33)
≥ Bachelor’s degree	4,366 (15)	1,328 (36)	5,694 (18)
Race (295 missing)
White	27,901 (98)	3,284 (88)	31,185 (97)
Other	640 (2)	134 (4)	774 (2)
BMI at time of survey (79 missing)
Underweight (BMI < 18.5 kg/m^3^)	414 (1)	38 (0.1)	452 (1)
Normal weight (BMI 18.5 to < 25.0 kg/m^3^)	7,693 (27)	972 (26)	8,665 (27)
Overweight (BMI 25.0 to < 30.0 kg/m^3^)	9,689 (34)	1,618 (44)	11,307 (35)
Obese (BMI ≥ 30.0 kg/m^3^)	10,694 (37)	1,057 (28)	11,751 (36)
Smoking
Never smoked	13,527 (47)	1,989 (54)	15,516 (48)
Smoked and quit	8,899 (31)	1,297 (35)	10,196 (32)
Smoked and did not quit	6,115 (21)	427 (12)	6,542 (20)
Regular alcohol consumption (74 missing)
Never	17,011 (60)	1,683 (45)	18,694 (58)
Yes and quit	4,105 (14)	535 (14)	4,640 (14)
Yes and did not quit	7,360 (26)	1,486 (40)	8,846 (27)
Any self-reported type 2 diabetes	3,912 (14)	522 (14)	4,434 (14)
Serum PFOA concentration measurement from the C8 Health Project (2005–2006)^*a*^ (ng/mL)
Mean	70.9	324.6	86.6
SD	151.2	920.6	278.9
25th percentile	12.3	55.9	12.8
Median	24.2	112.7	26.1
75th percentile	58.9	256.2	68.1
No. with measurements	28,422	1,881	30,303
^***a***^Frisbee et al. (2009).			

**Table 2:** Cardiovascular outcome frequencies (*n*).

Outcome	Reporting outcome	Reporting current medication or validated^*a*^ (% of reported)	Included in the primary retrospective analysis^*b*^	Included in the prospective analysis^*c*^
Hypertension	14,795	12,325 (83)	11,798	2,226
Hypercholesterolemia	15,825	9,909 (63)	9,653	1,825
Coronary artery disease	3,147	2,550 (81)^*a*^	2,468	515
^***a***^Validation was done only for coronary artery disease. Current medication includes self-reported use of current medication at the time of either survey round, or any medication use reported by a proxy. ^***b***^Had age at diagnosis and all other model covariates. ^***c***^Had age at diagnosis and all other model covariates, and age at diagnosis was after the age at the time of the C8 Health Project.				

**Figure 1:**
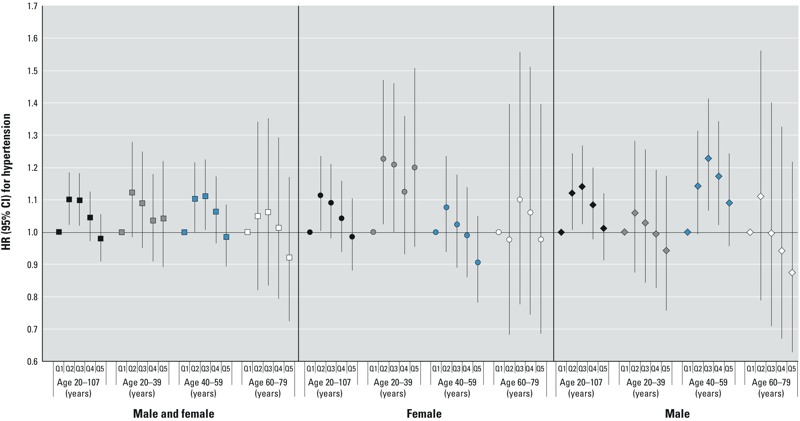
HRs and 95% CIs for hypertension in the primary retrospective analysis for the combined cohorts, cumulative exposure. Quintile (Q) cut points (μg/mL per year) were < 0.111, 0.111 to < 0.191, 0.191 to < 0.471, 0.471 to < 2.763, ≥ 2.763. The analysis included 11,798 cases of self-reported hypertension with medication. Models were stratified by single-year birth year and were either stratified by sex or controlled for sex and the interaction between sex and age. Models also controlled for years of schooling (not time-varying; < 12 years, high school diploma/GED, some college, or ≥ bachelor’s degree), race (white vs. nonwhite or missing), smoking (time-varying; current, former, none), smoking duration (time-varying), smoking pack-years (time-varying linear term created by multiplying the self-reported number of packs smoked per day by the smoking duration to that point), regular alcohol consumption (time-varying; current, former, none), BMI (at time of first study survey; underweight, normal, overweight, obese), and self-reported type 2 diabetes (time-varying according to reported age at diagnosis).

*Survival analysis results*. Hypertension. In the primary retrospective analysis, combining sexes and ages, there was no clear evidence of an association between cumulative PFOA exposure and hypertension [hazard ratios (HRs) and 95% confidence intervals (CIs) by increasing quintile: 1.00 (reference), 1.10 (95% CI: 1.02, 1.19), 1.10 (95% CI: 1.02, 1.18), 1.05 (95% CI: 0.97, 1.12), 0.98 (95% CI: 0.91, 1.06)] (Figure 1). Results of analysis using the yearly exposure metric were similar (data not shown). In age- and sex-stratified analyses (Figure 1), there was some evidence of increasing hypertension hazard with increasing cumulative or yearly exposure among females in the 20- to 39-year age group and males in the 40- to 59-year age group, but trends in hazard across exposure quintiles were not monotonic, and tests for trend using the logged exposure metrics were not statistically significant (at α = 0.05). Prospective analyses starting at the age in 2005–2006, and conditioning on absence of hypertension to that point, also showed no clear evidence of a positive association between cumulative (see Supplemental Material, Figure S3) or yearly PFOA exposure and hypertension, and showed HRs for some quintiles relative to quintile 1 that were < 1.

Retrospective analyses restricted to community cohort members had results similar to those of the primary analysis (see Supplemental Material, Figure S4); trends across PFOA exposure quintiles were not statistically significant (at α = 0.05). Results of retrospective analysis restricted to nonsmokers and analysis starting at the “qualifying age” were similar to those of the primary analysis (not shown). In retrospective analyses varying the year in which the analysis was ended, an apparent trend (although not completely monotonic) of increasing hypertension hazard with increasing cumulative PFOA exposure quintile was seen for sexes and ages combined in analyses ending in 1987; this trend became progressively less clear with longer follow-up after the time of peak exposure (see Supplemental Material, Figure S5).

**Figure 2:**
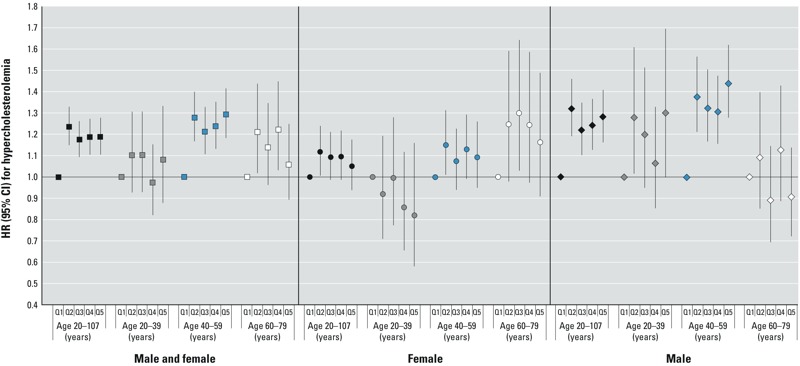
HRs and 95% CIs for hypercholesterolemia in the primary retrospective analysis for the combined cohorts, cumulative exposure. Quintile (Q) cut points (μg/mL per year) were < 0.142, 0.142 to < 0.234, 0.234 to < 0.630, 0.630 to < 3.579, ≥ 3.579. The analysis included 9,653 cases of self-reported hypercholesterolemia with medication. Models were stratified by single-year birth year and were either stratified by sex or controlled for sex and the interaction between sex and age. Models also controlled for years of schooling (not time-varying; < 12 years, high school diploma/GED, some college, or ≥ bachelor’s degree), race (white vs. nonwhite or missing), smoking (time-varying; current, former, none), smoking duration (time-varying), smoking pack-years (time-varying linear term created by multiplying the self-reported number of packs smoked per day by the smoking duration to that point), regular alcohol consumption (time-varying; current, former, none), BMI (at time of first study survey; underweight, normal, overweight, obese), and self-reported type 2 diabetes (time-varying according to reported age at diagnosis).

Hypercholesterolemia. The primary retrospective analysis, combining ages and sexes, showed an elevated hazard of hypercholesterolemia for exposure quintiles 2–5 relative to quintile 1 (Figure 2). For cumulative exposure, HRs increased in quintile 2 and remained elevated with little further increase across subsequent quintiles (HRs and 95% CIs by increasing quintile): 1.00 (reference), 1.24 (95% CI: 1.15, 1.33), 1.17 (95% CI: 1.09, 1.26), 1.19 (95% CI: 1.11, 1.27), 1.19 (95% CI: 1.11, 1.28) (test for trend using log cumulative exposure *p* = 0.005). Analyses using the yearly exposure metric also showed an increasing hazard of hypercholesterolemia with increasing quintiles (HRs and 95% CIs by increasing quintile): 1.00 (reference), 1.07 (95% CI: 1.01, 1.15), 1.11 (95% CI: 1.04, 1.19), 1.05 (95% CI: 0.99, 1.13), 1.20 (95% CI: 1.12, 1.28) (test for trend using log yearly exposure *p* < 0.001). In age and sex-specific analyses, the increased hazard for hypercholesterolemia was most pronounced among men 40–59 years of age (HRs and 95% CIs by increasing quintile): cumulative: 1.00 (reference), 1.38 (95% CI: 1.21, 1.56), 1.32 (95% CI: 1.17, 1.50), 1.31 (95% CI: 1.16, 1.48), 1.44 (95% CI: 1.28, 1.62) (test for trend using log cumulative exposure *p* < 0.001); yearly: 1.00 (reference), 1.16 (95% CI: 1.03, 1.30), 1.19 (95% CI: 1.05, 1.34), 1.16 (95% CI: 1.03, 1.31), 1.38 (95% CI: 1.23, 1.54) (test for trend using log yearly exposure *p* < 0.001). In analyses combining ages, tests for interaction between log exposure and sex were statistically significant (cumulative *p* = 0.048, yearly *p* = 0.004). Tests for interaction with age were also statistically significant (cumulative exposure, sexes combined, *p* = 0.015, males *p* = < 0.001; yearly exposure, males *p* = 0.032). Because of these interactions, analyses stratified by sex and age are preferred to the overall analyses. Prospective analyses starting at the age in 2005–2006 and conditioning on absence of hypercholesterolemia to that point showed no evidence of a positive association between hypercholesterolemia and higher PFOA exposure levels for the cumulative (see Supplemental Material, Figure S3) or yearly exposure metrics with Bayesian calibration (some observed HRs relative to quintile 1 were < 1), in analyses combining ages and sexes, or in analyses by age and sex (data not shown).

In retrospective analyses restricted to community cohort members, there was again evidence of a higher hazard of hypercholesterolemia with higher PFOA exposure in analyses combining sexes and ages (see Supplemental Material, Figure S6). Retrospective analyses restricted to nonsmokers (not shown) showed overall patterns of association similar to the primary analysis. Retrospective analyses starting at the “qualifying age” (not shown) also showed similar patterns of association, with significant tests for trend with log cumulative and yearly exposures in analyses combining ages and sexes, and for males 40–59 years of age. If analyses were ended in 1987, a strong trend of progressively increasing hazard of hypercholesterolemia with increasing PFOA exposure was seen in analyses combining ages and sexes, for both the cumulative (log-linear trend test *p* < 0.001) and yearly metrics (log-linear trend test *p* < 0.001) (see Supplemental Material, Figure S7 for yearly metric results). In analyses ending in progressively later years, the pattern of increasing hypercholesterolemia hazard with increasing PFOA exposure became progressively weaker for both exposure metrics.

**Figure 3:**
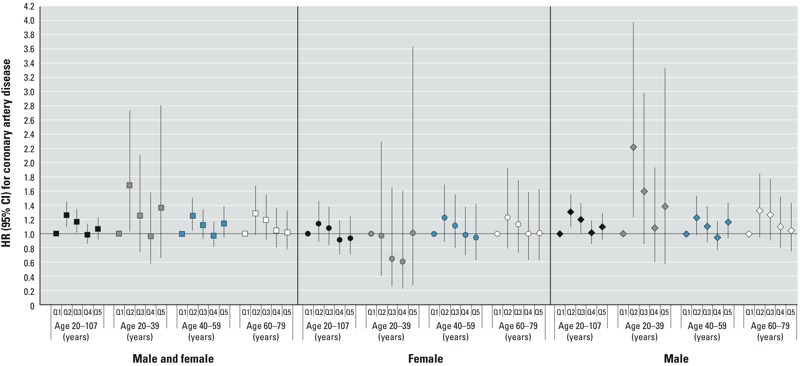
HRs and 95% CIs for coronary artery disease in the primary retrospective analysis for the combined cohorts, cumulative exposure. Quintile (Q) cut points (μg/mL per year) were < 0.147, 0.14 to < 0.248, 0.248 to < 0.717, 0.717 to < 5.058, ≥ 5.058. The analysis included 2,468 cases of validated coronary artery disease. Models were stratified by single-year birth year and were either stratified by sex or controlled for sex and the interaction between sex and age. Models also controlled for years of schooling (not time-varying; < 12 years, high school diploma/GED, some college, or ≥ bachelor’s degree), race (white vs. nonwhite or missing), smoking (time-varying; current, former, none), smoking duration (time-varying), smoking pack-years (time-varying linear term created by multiplying the self-reported number of packs smoked per day by the smoking duration to that point), regular alcohol consumption (time-varying; current, former, none), BMI (at time of first study survey; underweight, normal, overweight, obese), and self-reported type 2 diabetes (time-varying according to reported age at diagnosis).

Coronary artery disease. In the primary retrospective analysis, there was no clear association between PFOA exposure and coronary artery disease (Figure 3) (combining sexes and ages, HRs and 95% CIs by increasing quintile): cumulative 1.00 (reference), 1.26 (95% CI: 1.10, 1.45), 1.17 (95% CI: 1.02, 1.35), 0.99 (95% CI: 0.86, 1.14), 1.07 (95% CI: 0.93, 1.23); yearly 1.00 (reference), 1.02 (95% CI: 0.90, 1.16), 1.06 (95% CI: 0.93, 1.21), 0.95 (95% CI: 0.84, 1.09), 0.97 (95% CI: 0.85, 1.11). Among males 20–39 years of age, there appeared to be higher hazards of coronary artery disease in quintiles 2–5 relative to quintile 1, but CIs were wide for this age group, and there was no significant trend (log linear test for trend, cumulative exposure *p* = 0.64). Prospective analyses also showed no evidence of increasing hazards for coronary artery disease with increasing PFOA exposure, and showed HRs for quintiles 2–5 relative to quintile 1 that were often < 1 (see Supplemental Material, Figure S3).

In retrospective analyses restricted to community cohort members, patterns of associations were similar to those seen for the combined cohorts, with no clear associations (see Supplemental Material, Figure S8). Analyses restricted to nonsmokers and analyses starting at the “qualifying age” (not shown) were also similar. Analyses varying the year in which the analysis ended did not show clear monotonic trends across exposure quintiles, but had a suggestion of an association with coronary artery disease for cumulative and yearly PFOA exposure (for the sexes combined and among men) when the analysis was ended in years closer to peak PFOA exposures (see Supplemental Material, Figure S9, for yearly exposure results among men) (for analyses ending in 1987, log linear tests for trend: ages and sexes combined, cumulative *p* = 0.07, yearly *p* = 0.03; men, ages combined cumulative *p* = 0.11, yearly *p* = 0.061). Primary retrospective analysis for myocardial infarction also showed patterns of associations that were similar to those seen for coronary artery disease overall (data not shown).

## Discussion

In summary, there was little evidence of an increasing hazard of hypertension with increasing PFOA exposure, but there was evidence of an increasing hazard of hypercholesterolemia with increasing PFOA exposure, (most evident among males 40–59 years of age). Despite the observed association with hypercholesterolemia, there was no clear association between PFOA exposure and coronary artery disease. Although there was a suggestion of an association with coronary artery disease among males 20–39 years of age, CIs were wide for that group.

Our finding of an association between PFOA exposure and hypercholesterolemia is consistent with the findings of several previous studies. In humans, statistically significant positive associations between PFOA and serum cholesterol levels have been found in 10 studies (primarily cross-sectional) of people occupationally and non-occupationally exposed to PFOA (see “Introduction”); positive associations that were not statistically significant were found in four additional studies (Emmett et al. 2006; Olsen and Zobel 2007; Olsen et al. 2000; Wang et al. 2012). A longitudinal study of changes in serum cholesterol levels in relation to changes in serum PFOA levels, conducted in a subset of the same population considered in this study, found a small but significant association between decreases in serum PFOA concentrations and decreases in serum cholesterol levels (Fitz-Simon et al. 2013). A second longitudinal study of 179 workers involved with demolition of two former PFOA and PFOS manufacturing facilities (in Minnesota and Alabama), with a mean follow up of 164 days, did not find an association between changes in PFOA serum concentrations and changes in total cholesterol levels between a baseline assessment (before work on demolition) and an end-of-project assessment, either among the group overall or among the subgroup of 120 workers with baseline PFOA levels < 15 ng/mL and PFOS levels < 50 ng/mL (Olsen et al. 2012).

The evidence for associations between PFOA and either hypertension or coronary artery disease is sparse. One cross-sectional study of general population PFOA exposures, using data from NHANES, found an association between PFOA and systolic blood pressure and hypertension (Min et al. 2012). There has been little evidence of an association between PFOA exposure and coronary artery disease in previous occupational mortality studies of two cohorts (one of which considered the worker cohort in this study) (Lundin et al. 2009; Steenland and Woskie 2012). One cross-sectional study of PFOA exposure in the general U.S. population, using data from NHANES (1999–2000, 2003–2004, and 2005–2006) among people ≥ 20 years of age, also did not find an association between PFOA exposure and ischemic heart disease (defined as coronary artery disease, angina, and/or heart attack) (Melzer et al. 2010), but a second cross-sectional study of PFOA exposure using NHANES data (1999–2000 and 2003–2004), among people ≥ 40 years of age did find a statistically significant association with cardiovascular disease (defined as coronary artery disease, heart attack, or stroke) (Shankar et al. 2012).

The lack of an observed association between PFOA exposure and coronary artery disease in our study population may seem puzzling in light of the evidence for an association with hypercholesterolemia. As expected, time-varying hypertension and hypercholesterolemia were both strongly associated with an increased hazard of coronary artery disease in our study, after we controlled for the other variables in our models (results not shown). Nevertheless, effects of strong risk factors for coronary artery disease, such as smoking, could mask an indirect effect of PFOA operating through hypercholesterolemia as an intermediate outcome. However, PFOA also was not associated with coronary artery disease in a sensitivity analysis restricted to nonsmokers. In addition, treatment of hypercholesterolemia among our study participants might have mitigated potential effects of PFOA-associated hypercholesterolemia on coronary artery disease.

Strengths of this study include examination of a large cohort with exposure estimates going back to birth or to 1951, consideration of disease incidence (rather than mortality or prevalence), and validation of coronary artery disease diagnoses. This study also has several limitations. To be in the community cohort, a person had to be alive in 2005–2006, so some people who developed coronary artery disease may have been unavailable for enrollment in our study. Although workers did not have to be alive in 2005–2006, proxies were difficult to find for deceased workers. The impact on the analysis of loss of people who died of coronary artery disease is uncertain, because it is unclear whether this loss would be related to PFOA exposure. This study is also limited by the fact that exposure had been ongoing in this population for many years. If susceptibility to effects of PFOA on our outcomes varied in the population, this could result in progressively decreasing HRs with increasing follow-up time due to progressively decreasing susceptibility of the remaining population (Applebaum et al. 2011; Hernan 2010). We saw some evidence that this may have affected our results in analyses that ended in years closer to peak exposures. This issue may have particularly affected our prospective analyses (which were conditional on lack of disease before 2005–2006), and could explain prospective HRs relative to quintile 1 that were < 1.

Our primary analyses combined the community and worker cohorts to examine associations between PFOA and our outcomes across a wide exposure range. However, because workers had the highest exposures and may have been different from nonworkers in important ways, we considered sensitivity analyses restricted to nonworkers. Results of those analyses were generally similar to analyses including workers, suggesting that inclusion of workers did not led to substantial bias in our results.

Finally, there is a potential for misclassification of disease, exposure, and covariates because of inaccuracies in modeled exposure estimates and in self-reporting of disease and covariates. Any exposure misclassification would be expected to be independent of disease status and would likely, but not certainly, bias results toward the null. We sought to minimize misclassification of disease status by requiring prescription medication use for hypercholesterolemia and hypertension as well as medical records validation for coronary artery disease (to increase disease classification specificity), and by excluding from the analysis people who had some indication of disease, but did not meet the case definitions.

In conclusion, our study provided additional evidence supporting an association between PFOA exposure and hypercholesterolemia. However, we did not find strong evidence of an association between PFOA exposure and hypertension or coronary artery disease.

## Supplemental Material

(1 MB) PDF
