# Supplementary material for: Modeled PFOA Exposure and Coronary Artery Disease, Hypertension, and High Cholesterol in Community and Worker Cohorts
Source: Environ Health Perspect. 2014 Sep 26;122(12):1299–305. doi: 10.1289/ehp.1307943 (PMC4256699; doi:10.1289/ehp.1307943)
Supplement: (1 MB) PDF [file ehp.1307943.s001.508.pdf]

Environmental Health Perspectives doi:10.1289/ehp.1307943

Supplemental Material

Modeled PFOA Exposure and Coronary Artery Disease, Hypertension, and High Cholesterol in Community and Worker Cohorts

Andrea Winquist and Kyle Steenland

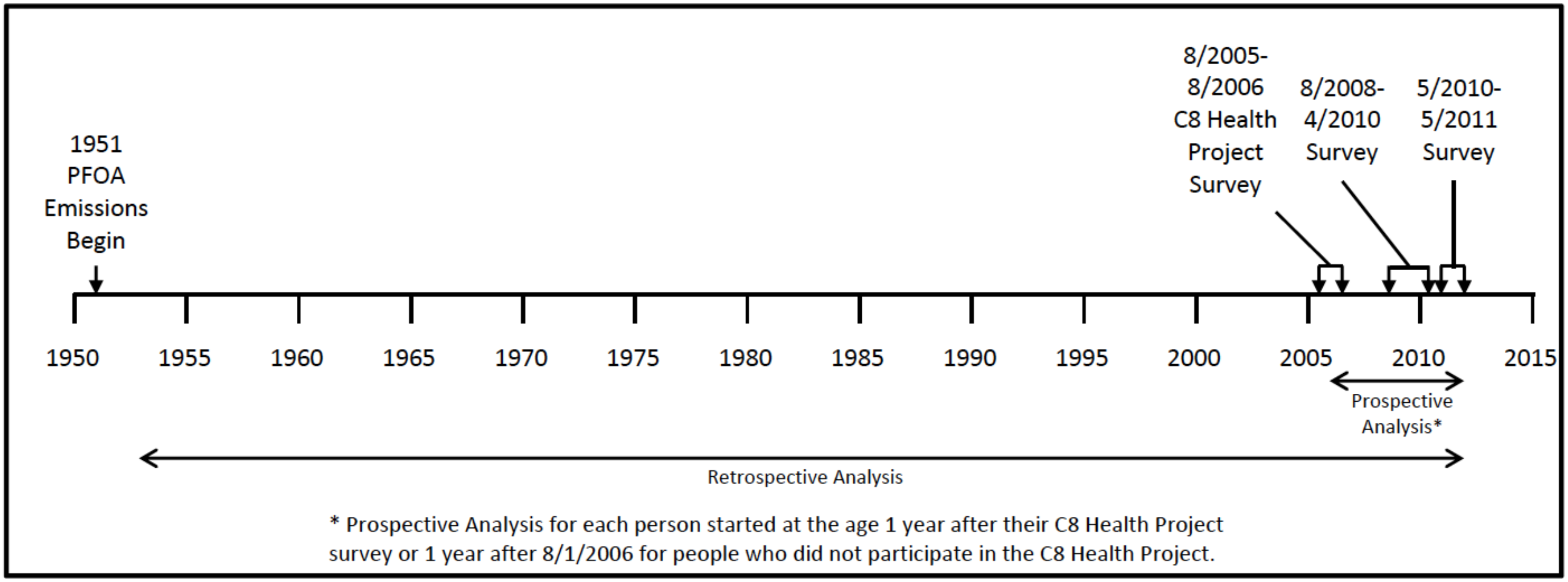

Figure S1. Timing of surveys and time periods covered by retrospective and prospective analyses.

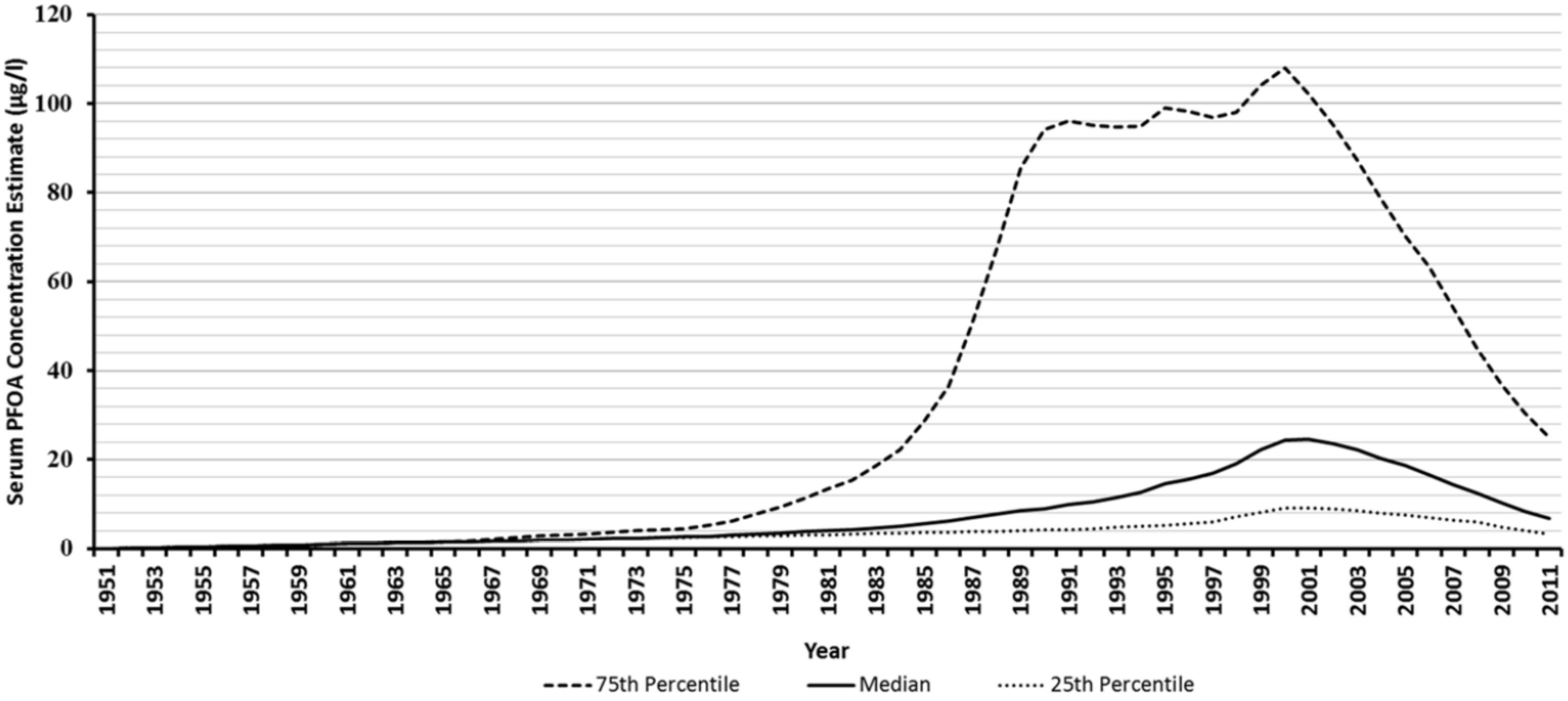

Figure S2. Retrospective Serum PFOA concentration estimates.

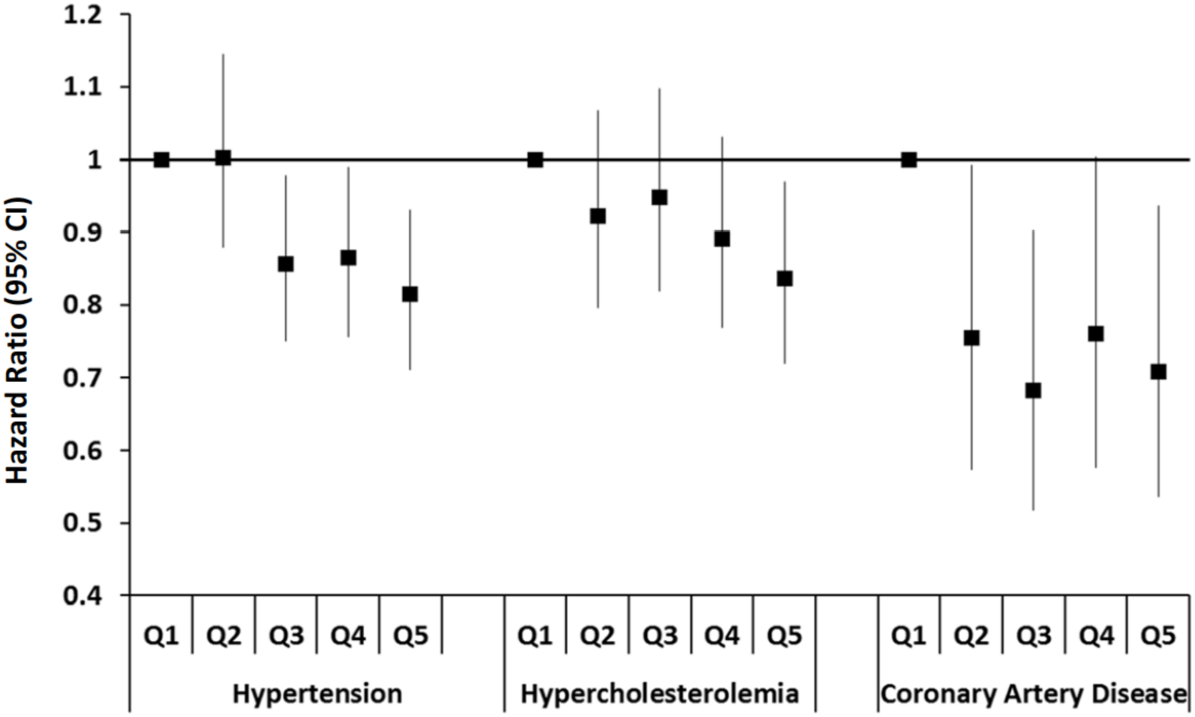

Figure S3. HR and 95% CI for prospective analysis, combined cohorts, cumulative exposure with Bayesian calibration, all ages, both genders. Quintile cut points (in µg/ml•yr) were: hypertension: <0.213, 0.213-<0.349, 0.349-<0.673, 0.673-<1.823, ≥1.823; hypercholesterolemia: <0.215, 0.215-<0.352, 0.352-<0.656, 0.656-<1.763, ≥1.763; coronary artery disease: <0.218, 0.218-<0.396, 0.396-<0.775, 0.775-<2.143, ≥2.143. Models were stratified by single-year birth year and controlled for gender and the interaction between gender and age. Models also controlled for years of schooling (not time-varying; <12 years, high school diploma/GED, some college, or bachelor's degree or higher), race (white vs. non-white or missing), smoking (time-varying; current, former, none), smoking duration (time varying), smoking pack years (time-varying linear term created by multiplying the self-reported number of packs smoked per day by the smoking duration to that point), regular alcohol consumption (time-varying; current, former, none), BMI (at time of first study survey; underweight, normal, overweight, obese), and self-reported type 2 diabetes (time-varying according to reported age at diagnosis).

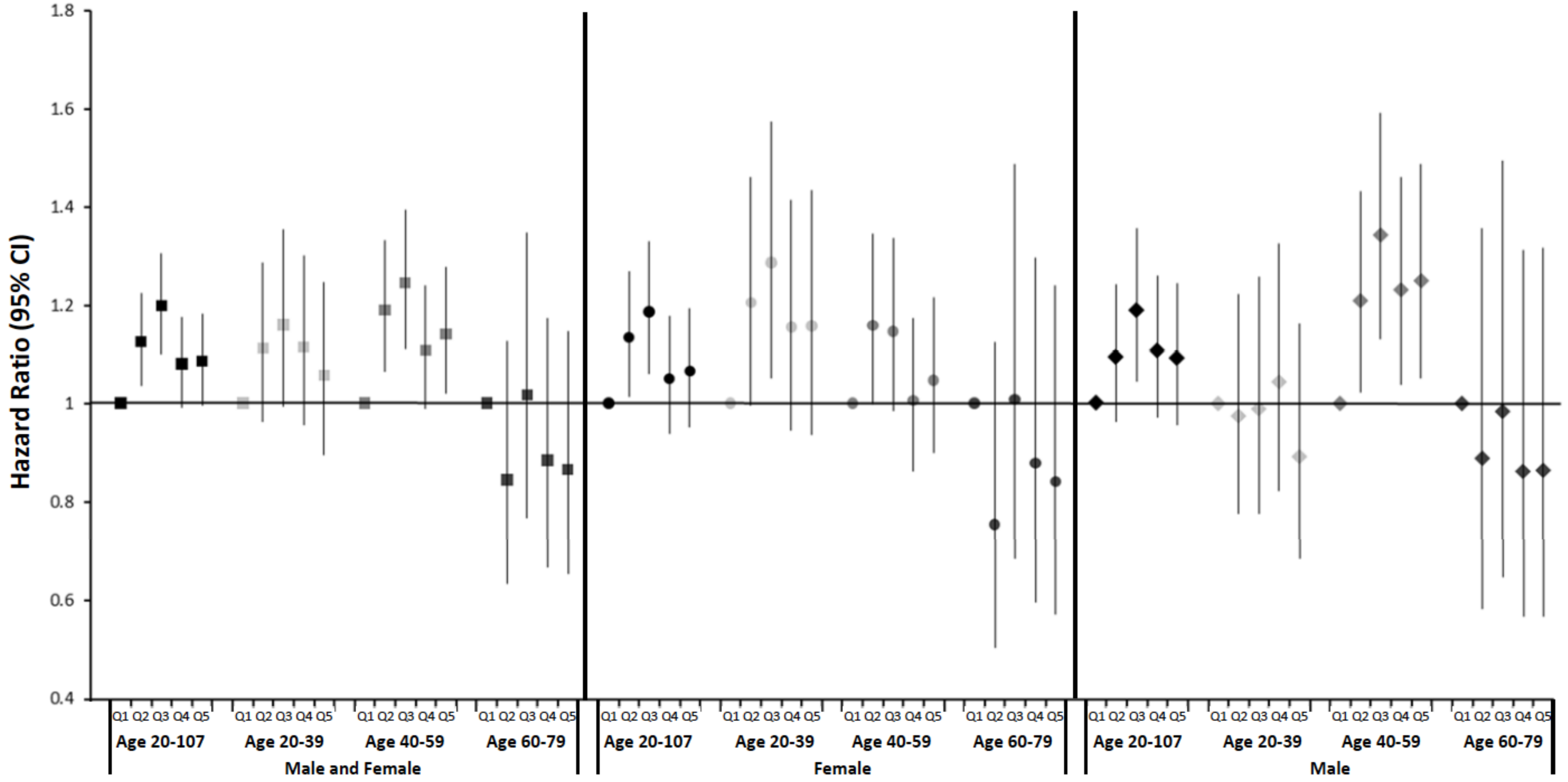

Figure S4. HR and 95% CI for hypertension, retrospective analysis, cumulative exposure, community cohort only. Models were stratified by single-year birth year and were either stratified by gender or controlled for gender and the interaction between gender and age. Models also controlled for years of schooling (not time-varying; <12 years, high school diploma/GED, some college, or bachelor's degree or higher), race (white vs. non-white or missing), smoking (time-varying; current, former, none), smoking duration (time varying), smoking pack years (time-varying linear term created by multiplying the self-reported number of packs smoked per day by the smoking duration to that point), regular alcohol consumption (time-varying; current, former, none), BMI (at time of first study survey; underweight, normal, overweight, obese), and self-reported type 2 diabetes (time-varying according to reported age at diagnosis).

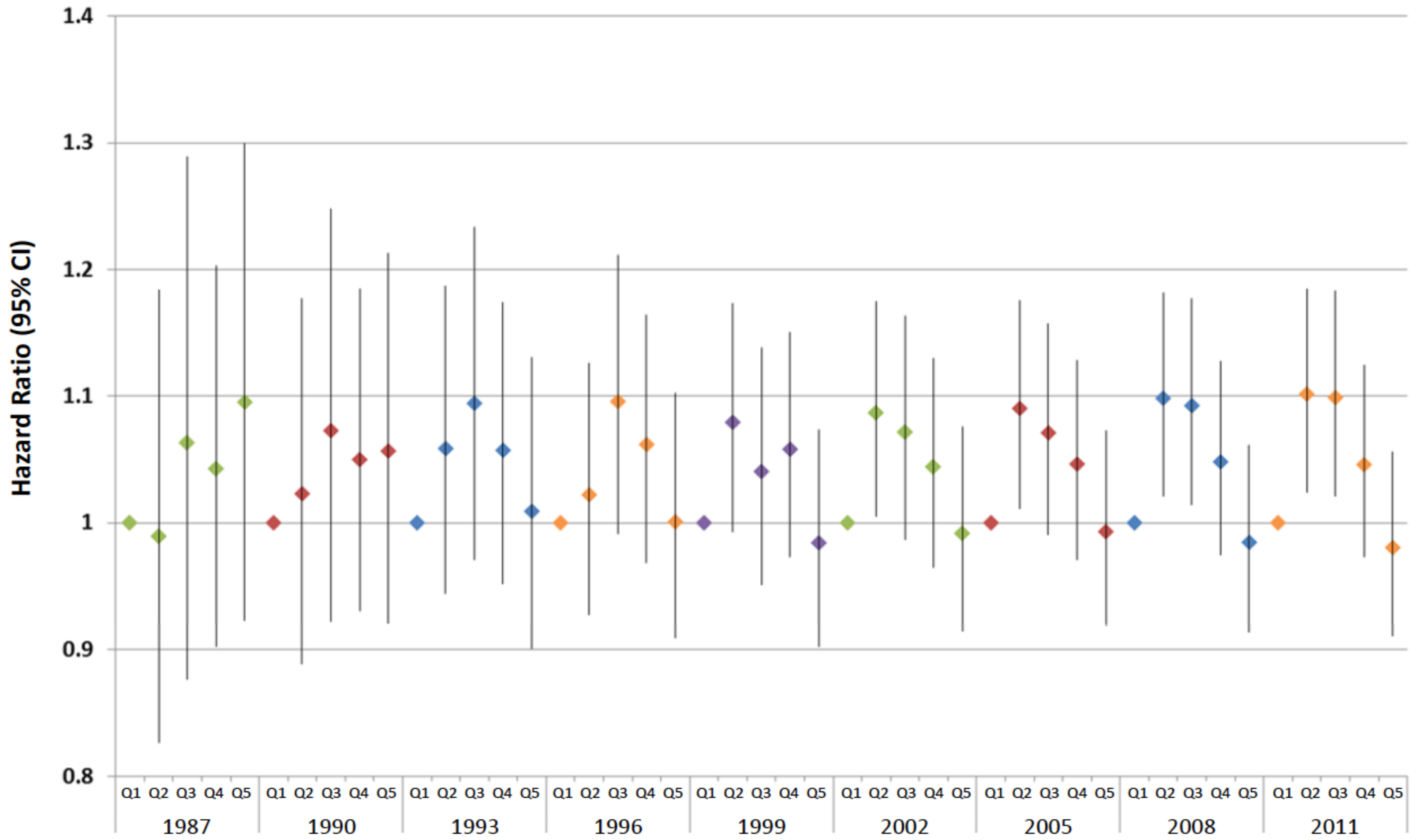

Figure S5. HR and 95% CI for hypertension, retrospective analysis, cumulative exposure, combined cohorts, both genders, all ages, varying end year. Models were stratified by single-year birth year and controlled for gender and the interaction between gender and age. Models also controlled for years of schooling (not time-varying; <12 years, high school diploma/GED, some college, or bachelor's degree or higher), race (white vs. non-white or missing), smoking (time-varying; current, former, none), smoking duration (time varying), smoking pack years (time-varying linear term created by multiplying the self-reported number of packs smoked per day by the smoking duration to that point), regular alcohol consumption (time-varying; current, former, none), BMI (at time of first study survey; underweight, normal, overweight, obese), and self-reported type 2 diabetes (time-varying according to reported age at diagnosis).

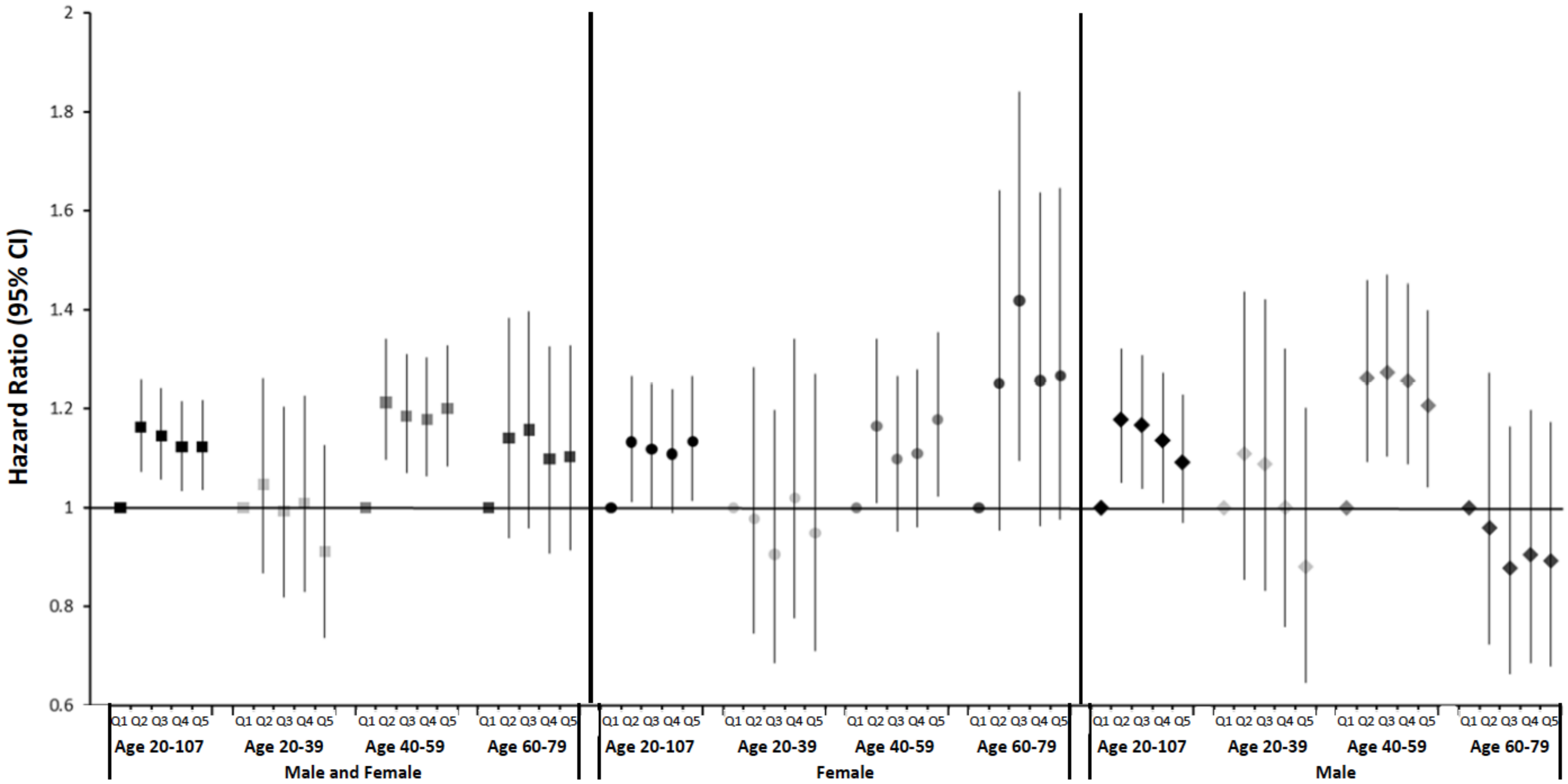

Figure S6. HR and 95% CI for hypercholesterolemia, retrospective analysis, cumulative exposure, community cohort only. Models were stratified by single-year birth year and were either stratified by gender or controlled for gender and the interaction between gender and age. Models also controlled for years of schooling (not time-varying; <12 years, high school diploma/GED, some college, or bachelor's degree or higher), race (white vs. non-white or missing), smoking (time-varying; current, former, none), smoking duration (time varying), smoking pack years (time-varying linear term created by multiplying the self-reported number of packs smoked per day by the smoking duration to that point), regular alcohol consumption (time-varying; current, former, none), BMI (at time of first study survey; underweight, normal, overweight, obese), and self-reported type 2 diabetes (time-varying according to reported age at diagnosis).

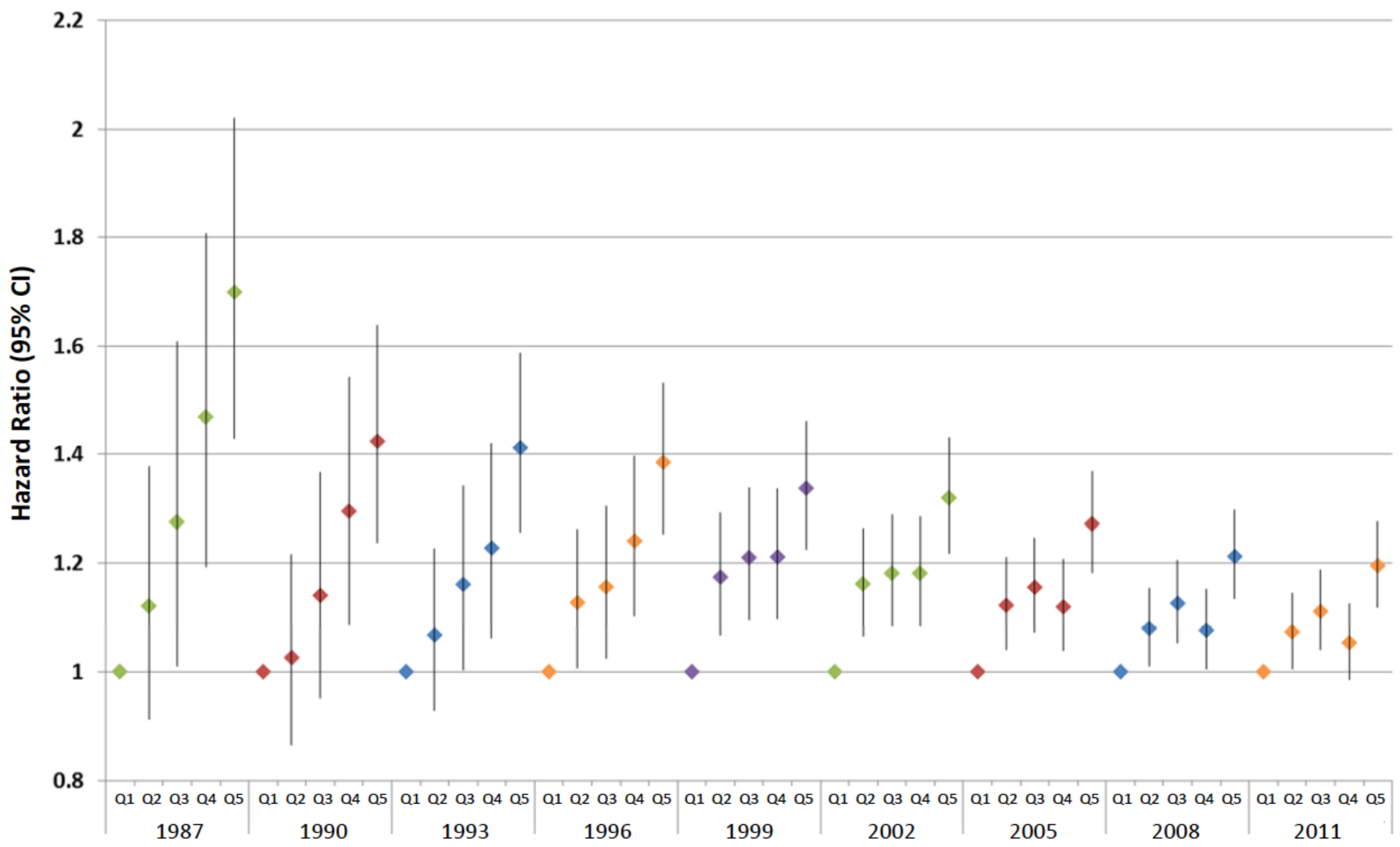

Figure S7. HR and 95% CI for hypercholesterolemia, retrospective analysis, yearly exposure, combined cohorts, both genders, all ages, varying end year. Models were stratified by single-year birth year and controlled for gender and the interaction between gender and age. Models also controlled for years of schooling (not time-varying; <12 years, high school diploma/GED, some college, or bachelor's degree or higher), race (white vs. non-white or missing), smoking (time-varying; current, former, none), smoking duration (time varying), smoking pack years (time-varying linear term created by multiplying the self-reported number of packs smoked per day by the smoking duration to that point), regular alcohol consumption (time-varying; current, former, none), BMI (at time of first study survey; underweight, normal, overweight, obese), and self-reported type 2 diabetes (time-varying according to reported age at diagnosis).

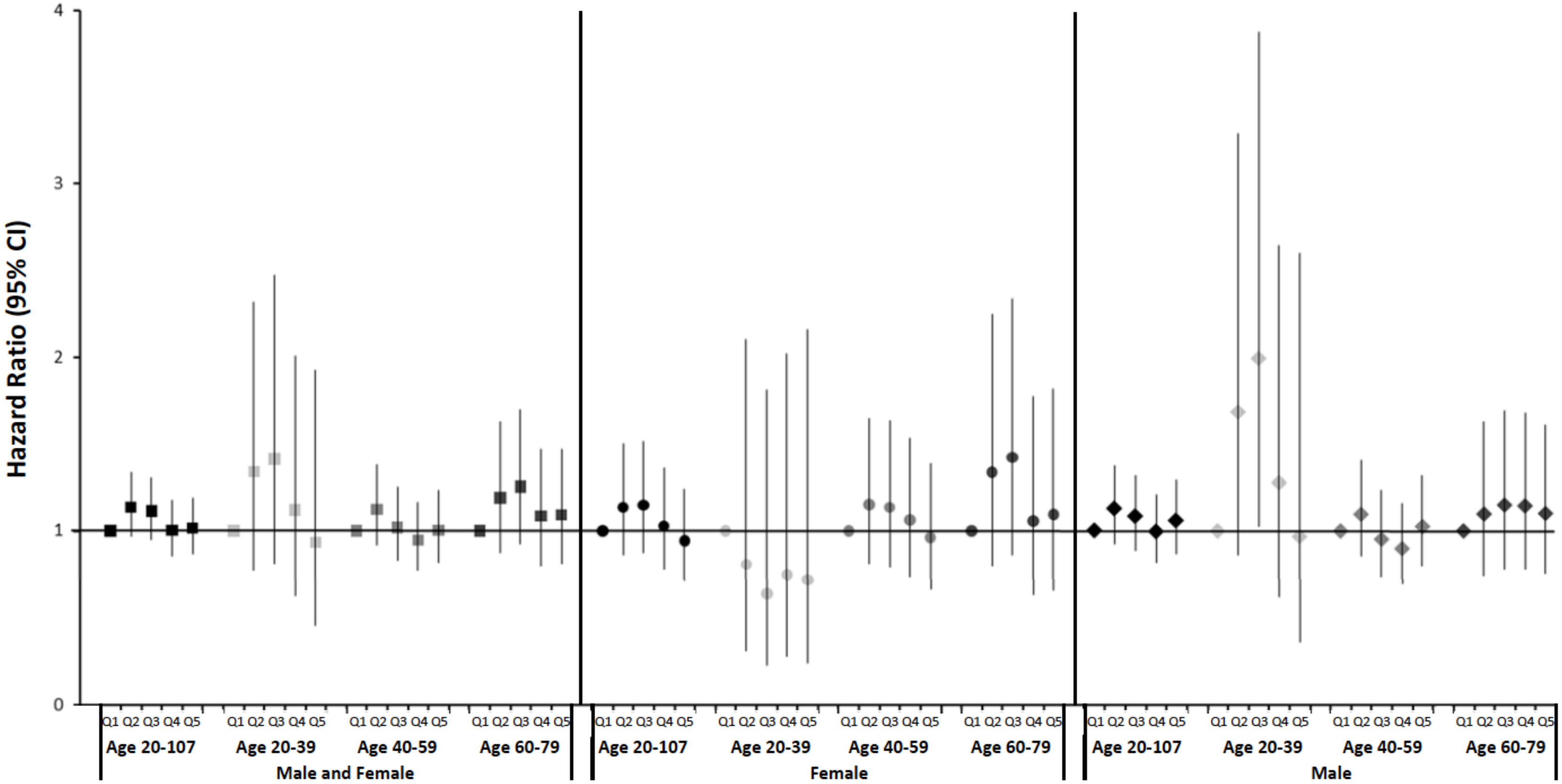

Figure S8. HR and 95% CI for coronary artery disease, retrospective analysis, cumulative exposure, community cohort only. Models were stratified by single-year birth year and were either stratified by gender or controlled for gender and the interaction between gender and age. Models also controlled for years of schooling (not time-varying; <12 years, high school diploma/GED, some college, or bachelor's degree or higher), race (white vs. non-white or missing), smoking (time-varying; current, former, none), smoking duration (time varying), smoking pack years (time-varying linear term created by multiplying the self-reported number of packs smoked per day by the smoking duration to that point), regular alcohol consumption (time-varying; current, former, none), BMI (at time of first study survey; underweight, normal, overweight, obese), and self-reported type 2 diabetes (time-varying according to reported age at diagnosis).

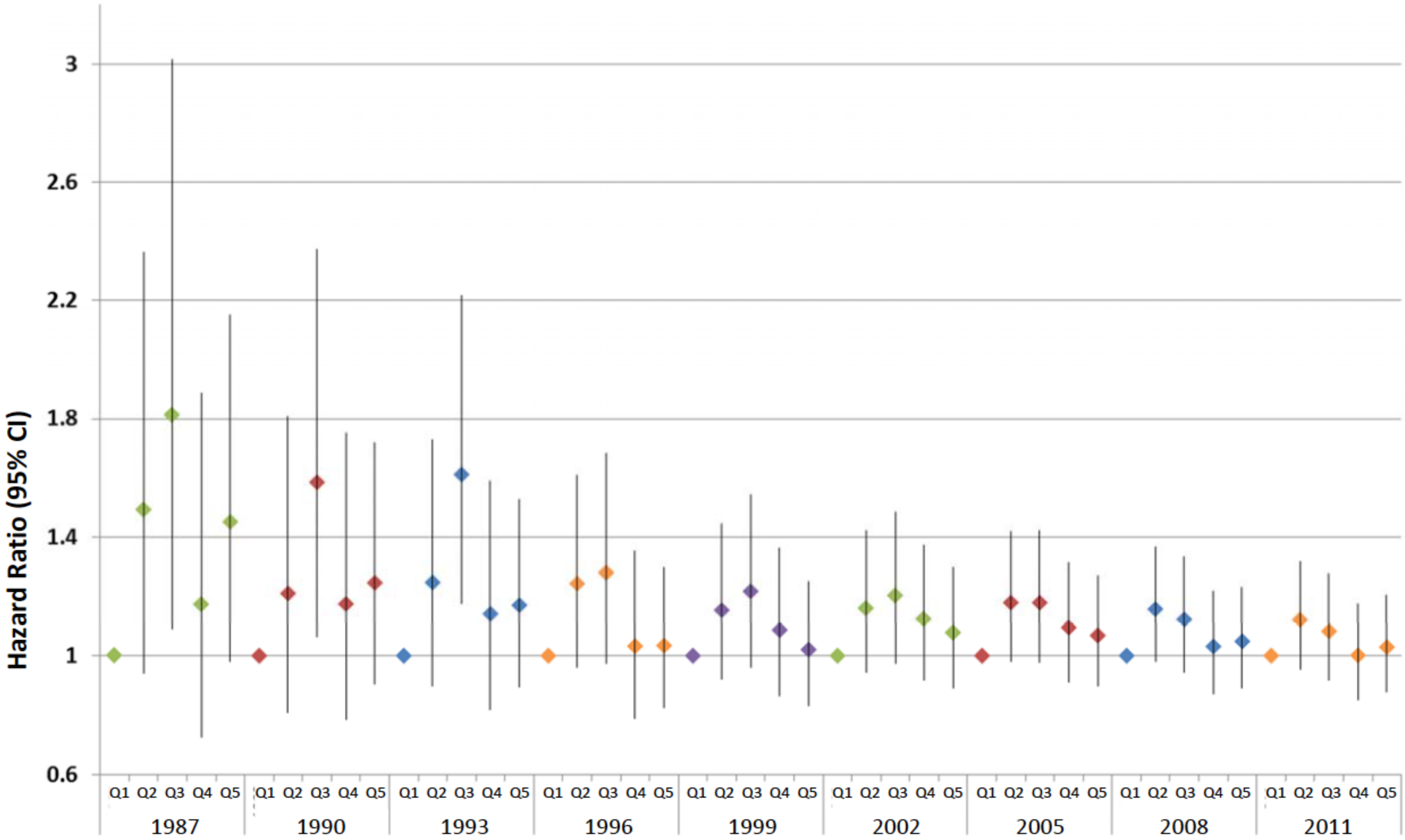

Figure S9. HR and 95% CI for coronary artery disease, retrospective analysis, yearly exposure, combined cohorts, men only, all ages, varying end year. Models were stratified by single-year birth year and were restricted to men. Models controlled for years of schooling (not time-varying; <12 years, high school diploma/GED, some college, or bachelor's degree or higher), race (white vs. non-white or missing), smoking (time-varying; current, former, none), smoking duration (time varying), smoking pack years (time-varying linear term created by multiplying the self-reported number of packs smoked per day by the smoking duration to that point), regular alcohol consumption (time-varying; current, former, none), BMI (at time of first study survey; underweight, normal, overweight, obese), and self-reported type 2 diabetes (time-varying according to reported age at diagnosis).
